# Perceptions of risk and motivation for healthy living among immigrants from non-western countries with prior gestational diabetes mellitus living in Denmark

**DOI:** 10.1080/21642850.2021.1969235

**Published:** 2021-08-30

**Authors:** Stine Bagger, Helle Terkildsen Maindal, Karoline Kragelund Nielsen, Amanda Grønbjerg Vrå, Jens Aagaard-Hansen

**Affiliations:** a Health Promotion Research, Steno Diabetes Center Copenhagen, Gentofte, Denmark; b Department of Public Health, Section for Health Promotion, Aarhus University, Aarhus, Denmark; c Faculty of Health Sciences, SA MRC Developmental Pathways for Health Research Unit, University of the Witwatersrand, Johannesburg, South Africa

**Keywords:** Ethnicity, gestational diabetes, motivation, risk perception, migration

## Abstract

**Objective:**

To explore perceptions of risk and motivation for healthy living among immigrant women from non-western countries with prior gestational diabetes mellitus (GDM) living in Denmark.

**Design:**

Seventeen semi-structured interviews were conducted with 12 female immigrants with prior GDM from non-western countries living in Denmark. The women were recruited through a public hospital and other health services and nongovernmental organisations. The theoretical approach was inspired by Arthur Kleinman’s Explanatory Models. Data were analysed using qualitative content analysis.

**Results:**

A diagnosis of GDM entailed great worry for the future. Participants’ fears were primarily linked to the potential later development of type 2 diabetes (T2D) and poor health. Women’s perceptions of GDM reflected their experiences with T2D-related complications and even death among relatives. The risk perception of GDM was also influenced by participants’ challenges and trauma unrelated to diabetes. Their motivation for healthy living was strengthened by their experiences with T2D among relatives, while unrelated challenges and trauma generally reduced their capacity for healthier behaviours.

**Conclusion:**

Among women with a non-western immigrant background and prior GDM living in Denmark, experiences with T2D among family members and their close communities affect their perceptions of risk and motivation to prevent the development of T2D. Furthermore, the challenges of daily life and past trauma were critical factors in their levels of available resources for health. Health promotion in this population should address health in a holistic way by integrating mental and social health with interventions aimed at preventing the development of T2D.

## 1. Introduction

### 1.1 Gestational diabetes mellitus as a public health challenge

Gestational diabetes mellitus (GDM) is defined by the American Diabetes Association as diabetes diagnosed in the second or third trimester of pregnancy that was not clearly overt diabetes prior to gestation (American Diabetes Association, 2020). In most cases, GDM is a transient form of diabetes that resolves after delivery. It affects 2–6% of pregnancies in Europe, although detection practices vary (Eades, Cameron, & Evans, 2017). In Denmark, GDM is diagnosed in approximately 3% of pregnancies that result in delivery (Jeppesen, Maindal, Kristensen, Ovesen, & Witte, 2017). Detecting and managing GDM is important because it is associated with an increased risk of adverse outcomes for mother and child that include obstructed labour, pre-eclampsia, pre-term delivery, macrosomia and shoulder dystocia (Black, Sacks, Xiang, & Lawrence, 2010; Metzger et al., 2008; Waters et al., 2016).

Furthermore, women with previous GDM have a more than sevenfold increased risk of developing diabetes (primarily type 2 diabetes [T2D]) and an increased risk of future cardiovascular disease, compared to women with normoglycemic pregnancies (Bellamy, Casas, Hingorani, & Williams, 2009; Kramer, Campbell, & Retnakaran, 2019). In addition, the hyperglycaemic intrauterine environment places the offspring at increased long-term risk for metabolic disorders (Clausen et al., 2008, 2009). Follow-up studies have shown that offspring exposed to GDM in utero are more likely to have general and central obesity and exhibit insulin resistance and glucose intolerance in early adulthood (Metzger, 2007).

Studies have shown that the risk of T2D can be reduced in high-risk groups, including women with previous GDM (Ratner et al., 2008). Results from the American Diabetes Prevention Programme show that lifestyle interventions reduced progression of T2D over 10 years by 35% among women with previous GDM (Aroda et al., 2015). Thus, it is recommended that women with a history of GDM are monitored and follow a healthy lifestyle, although studies suggest that this is often not the case (Nielsen, Kapur, Damm, De Courten, & Bygbjerg, 2014; Stage, Ronneby, & Damm, 2004).

Women who are immigrants from other countries may be at particular risk of GDM. A recent Danish study documented that women immigrating from various countries in Asia, the Middle East and North Africa have a two to fivefold increased risk of GDM, compared to women born in Denmark, and almost one-quarter of all GDM diagnoses in Denmark are among immigrant women (Nielsen, Andersen, Damm, & Andersen, 2020). Consequently, it is important to understand the perceptions, challenges, and previous experiences of GDM among immigrant women to prevent the future development of T2D.

The study aim was to explore perceptions of risk and motivation for healthy living among immigrant women from non-western countries with previous GDM living in Denmark.

### 1.2 Management and long-term follow-up of GDM in Denmark

In Denmark, women with GDM are usually diagnosed as part of antenatal care services. According to Danish national recommendations (Danish Health Authority, accessed August 22, 2020), women diagnosed with GDM are offered self-management education on controlling blood glucose levels during pregnancy with a healthy diet and increased physical exercise. They also receive instructions on using a glucose metre to monitor their blood glucose levels. The first week they are instructed to measure their blood glucose four times each day, 90 minutes before and after breakfast and dinner. If blood glucose levels are stable, measurement frequency decreases to two days a week. If dietary changes and physical activity fail to normalise blood glucose levels, insulin treatment is initiated.

Due to the high risk of future diabetes, women with GDM are advised to have an oral glucose tolerance (OGTT) test within three to four months of delivery and HbA1c tests performed at least every three years by their general practitioner (ibid.).

## 2. Material and methods

### 2.1 Study design and sampling

The qualitative design was based on interviews conducted in Copenhagen, Denmark, between December 2018 and February 2020. Women with previous GDM and a non-western immigrant background were eligible to participate. To ensure maximum variation, women representing different age groups, educational levels and countries of origin were purposively recruited, regardless of Danish proficiency. Women diagnosed with T2D were excluded. Sampling continued until no new themes emerged, indicating data saturation.

Recruitment took place during pregnancy or the waiting period for the postpartum OGTT at the obstetric departments at two large hospitals in the Copenhagen area. In addition, women were recruited through a municipal diabetes centre in Copenhagen and nongovernmental organisations working with the target group. Identifying potential participants proved somewhat challenging, necessitating the use of multiple recruitment sites. Typically, healthcare professionals approached potential participants and asked if they would like to participate; if they agreed, the researcher subsequently contacted them. Only a few women declined participation, primarily due to mental health issues. All participants gave birth in Denmark.

### 2.2 Data collection methods

A total of 17 semi-structured in-depth interviews were conducted with 12 non-western immigrant women with prior GDM. Three women were interviewed twice, and one woman was interviewed three times. Interviews lasted 30–110 minutes and took place at hospitals, libraries, a municipal diabetes centre or took place at participants’ homes. Four interviews were conducted in English due to participants’ lack of Danish proficiency. Interpreters for other languages were available but not needed.

The semi-structured interview guide comprised questions such as: What crossed your mind when receiving the GDM diagnosis? Does your previous GDM affect you in any way now? The interview guide also included questions regarding daily life, habits and any intended behaviour changes such as: Please mention five examples of what you consider a healthy life? What do you eat during a typical day (e.g. yesterday)? What is healthy food for you? Does your previous GDM diagnosis affect your and your family’s lifestyle? Would you like to live healthier than you do now? If so, how?

During interviews, participating women were asked to complete a sociogram, a visual tool illustrating a person’s relationships and the degree of their importance (Hennig, Brandes, Pfeffer, & Mergel, 2012; Ryan, Mulholland, & Agoston, 2014). Participating women were also asked to mark major life events on a timeline (Adriansen, 2012) and explain the significance of each event. The sociogram and timelines provided opportunities to understand their narratives, experiences and perceptions in a broader context. The researchers also used written field notes throughout the study to capture participants’ nonverbal communication and relevant contextual factors.

### 2.3 Theoretical framework

The study was inspired by Arthur Kleinman’s (1980) explanatory model, which provides a theoretical framework for understanding how patients understand illness, including aetiology, time and mode of onset of symptoms, pathophysiology, course of sickness and treatment. Explanatory models are used to compare different systems of knowledge and values within the healthcare system, such as relationships between patients and healthcare practitioners, where lay perspectives (the emic ‘illness’) and professional perspectives (the etic ‘disease’) meet. The study aim was to understand participants’ perception of risk and motivation for healthy living after delivery, so we used only the parts of the theoretical framework that supported this aim, omitting those addressing relationships between participants and healthcare providers.

To understand perceptions of risk and motivation for healthy living, we paid particular attention to the variety of contextual challenges faced by participating women, supplementing the explanatory model with Kleinman’s (1997) later work on experience and suffering.

### 2.4 Data analysis

Interviews, including discussions about sociograms and timelines, were audio-recorded, transcribed and analysed using NVivo (version 12, QSR International), following deductive and inductive processes. Data were analysed using thematic analysis (Braun & Clarke, 2006), based on a combination of deduction and induction. The main categories of Kleinman’s explanatory models led to several deductive coding categories, and a supplementary inductive approach captured new themes not previously imagined by the researchers. An initial read through of the transcripts provided an overview of the data and familiarity with transcribed interviews. Transcripts were then re-read and coded, after which overarching themes were identified. Field notes were read, re-read, coded, and included in the thematic analysis as described.

### 2.5 Ethical considerations

The study adhered to all national and institutional regulations. According to the Danish Regional Ethical Committee, specific ethical clearance was not required, whereas the project was registered at The Danish Data Protection Agency (journal number: VD-2018-192). The study meticulously followed the principles of the Council for International Organizations of Medical Sciences including informed consent, nonmaleficence, confidentiality and beneficence (CIOMS, 2016). Thus, before interviews, participating women signed a statement of informed consent that included information about the study purpose, anonymity and voluntary participation, as well as the option to withdraw from the study without any repercussions.

## 3. Results

### 3.1 Characteristics of study participants

A total of 12 women were interviewed (Table 1). They had immigrated from countries in Africa, the Middle East or Asia and were 29–52 years of age at the time of their interview(s). Elapsed time since the most recent pregnancy with a GDM diagnosis was 10 weeks to 20 years. The findings did not vary by duration of stay in Denmark or time since the most recent GDM pregnancy.

**Table 1.** Characteristics of the informants in terms of country of origin, age, years in Denmark, time since GDM diagnosis, religion, occupation, number of children and total number of pregnancies with a GDM diagnosis at the time of the interview.

Participant	Country of origin	Age	Years in Denmark	Time since last pregnancy with a GDM diagnosis	Religion	Occupation	Number of children (Total number of pregnancies with a GDM diagnosis)
1	Kurdistan, Iraq	42	23	3 years	Muslim	Job	4 (1)
2	Pakistan	35	27	11 months	Muslim	On leave with job	4 (1)
3	Iraq	52	20	16 years	Muslim	Job	3 (1)
4	Somalia	35	21	3 years	Muslim	Job	5 (1)
5	Iraq	51	26	20 years	Muslim	No job	3 (1)
6	Pakistan	35	20	3 months	Muslim	On leave with job	2 (2)
7	India	39	2	10 weeks	Hindu	On leave with job	1 (1)
8	China	40	26	2 months	Orthodox Christian	On leave with job	4 (4)
9	India	36	8	3 months	Hindu	On leave with job	1 (1)
10	Morocco	48	47	14 years	Muslim	Job training	2 (1)
11	Lebanon/Palestine	29	17	4 months	Muslim	Student	1 (1)
12	Pakistan	30	9	4 months	Muslim	No job	2 (1)

### 3.2. Perceptions of risk and motivation for healthy living

Four themes were derived from Kleinman’s (1980) explanatory model: (1) aetiology, (2) symptoms, (3) risk and prognosis and (4) management and pathophysiology. A fifth theme emerged from the inductive analysis: (5) contextual challenges.

#### 3.2.1 Aetiology

Generally, participants mentioned genetic susceptibility as the primary reason for their previous diagnosis of GDM. Most had close relatives diagnosed with T2D.

It’s just a gene that I got from my parents. (participant 6)

Because it runs in the family. My mother had it. All her siblings had it. My elder brother has it. My elder sister has it. (participant 2)

However, genetic susceptibility was not the only reason participants gave for their diagnosis. Overweight and diet were also common and important explanations for GDM. In particular, traditional food and cooking habits were perceived as contributing factors.

But it is just our habits, because when we pour oil, then we don’t measure [the amount of oil]. We just pour from the bottle … . I think it is our food. Plus, we Pakistanis really drink a lot of milk. (participant 2)

Moreover, some participants had experienced specific cravings in the early stages of pregnancy and linked them to the later development of GDM.

I think I ate a lot of sugar during my pregnancy. I remember that I took a glass of water with lemon 2–3 months into my pregnancy and added two spoonfuls of sugar. I drank it all the time. … .. I thought that was why I got GDM. In my other pregnancies I did not do like that. (participant 4)

One participant mentioned God’s will as a factor in developing GDM. However, despite the acknowledged power of God, this participant still considered herself capable of changing habits to delay what might be meant to follow. Other contributing causes to GDM described by participants were pregnancy at a relatively old age and psychological distress. Sedentary behaviour was mentioned only briefly by a single participant as a contributing factor.

#### 3.2.2 Symptoms

Participants found it hard to distinguish between GDM-specific symptoms and general symptoms of pregnancy, especially if it was a first pregnancy or they had had GDM during all previous pregnancies. Even women with previous normoglycemic pregnancies could be in doubt.

But so many things happen during pregnancy. You don’t know if [signs or symptoms] are related to this or that [pregnancy or GDM]. (participant 1)

Only one woman described noticing a difference in symptoms between previous normoglycemic pregnancies and a pregnancy with GDM.

I was tired. I did not want to do anything, and when I got hungry, I started to shake like this [demonstrates]. And I had no power. I wanted to rest in bed … I tell you, with my fifth child, I felt bad. I didn’t sleep much, and I was very ill, and I got diabetes and I vomited. … . It is the most difficult pregnancy I have had with my children. (participant 4)

Thus, for most participating women, a diagnosis of GDM was ‘invisible’. Symptoms were hard to detect and distinguish from symptoms of pregnancy itself.

#### 3.2.3 Risk and prognosis

Participants described their reactions to being diagnosed with GDM as primarily ones of shock and despair. Several women reported that they had thought of themselves as too young for a diabetes-related diagnosis. For some participants, the diagnosis also generated concerns that diabetes would develop into a chronic condition such as T2D or that their child in some way had been harmed during pregnancy.

All participants were aware that a GDM diagnosis was associated with an increased risk of developing T2D later in life. Even though they found it reassuring that GDM was a temporary condition, the primary concern during pregnancy for most participants was the risk of developing T2D later in life.

It was a really difficult period. I was really worried whether I would get diabetes afterwards. (participant 3)

Participants’ concerns about developing T2D persisted after delivery. Yet, all women were aware that engaging in specific health behaviours could have a preventative effect.

It really means a lot after delivery, because I have to be careful, and my midwife has said that if I start [eating unhealthy food] again there is a very big risk that I’ll get diabetes. (participant 4)

Participants’ knowledge of T2D and its course and implications were heavily influenced by their experiences of relatives and friends with T2D. Many women had experienced family members with T2D as struggling to follow the recommended diet, and they described diabetes as very detrimental and requiring a restrictive lifestyle.

Well, I got upset … Like, will I have to live like this the rest of my life? … and eat food like this? … . Constantly thinking ‘oh, I can’t eat this because I’ve got diabetes’. (participant 2)

Many participants had relatives or friends for whom T2D had been severe or even deadly. They were highly aware of possible adverse outcomes and complications.

What makes me nervous about diabetes is if you get wounds … . that it is difficult to heal. And that makes me scared … . Because I have seen my uncle lose his big toe. (participant 10)

Participants described their awareness of T2D challenges and outcomes as significantly influencing their motivation for preventative behaviours.

Yes, of course I would like to live a healthier life … Because there are so many issues, for instance, as I say, in my family there is diabetes and cholesterol. And my father got a coronary thrombosis. … .. So I think, you know what? … . I would like to do something about it. (participant 6)

Participants were aware of their own increased risk of developing T2D and their experiences with relatives with T2D-related complications. Furthermore, these factors increased their motivation to maintain health-promoting habits formed during pregnancy while following GDM self-management recommendations. Thus, participants’ awareness of potential consequences of a future T2D diagnosis affected their perception of risk and motivation for healthy living.

#### 3.2.4 Management and pathophysiology

Among participating women, biomedical knowledge about GDM was generally limited. Some women could explain some biomedical aspects, and others seemed somewhat confused and embarrassed when they realised they could not give an explanation.

Is it not that. … I am not quite sure. But there are some hormones that change … . I don’t know. (participant 1)

However, participants’ limited biomedical knowledge did not affect their understanding of GDM self-management guidelines. In general, they described feeling well informed and well equipped to follow the recommendations they had been given.

And if I thought, OK, for example, eeehh, I have eaten … Pakistani pancakes [with a lot of sugar] … the sugar will probably increase … . I knew that my sugar level was probably 8 or 9, so I just went for a walk. (participant 6)

A single participant was being managed with insulin during pregnancy. The others managed their blood glucose levels with changed diet and exercise patterns. They all described GDM self-management as a radical change to their everyday lives that had been challenging to implement. They had had to reduce or eliminate food items that were otherwise the main components of their usual diet, such as white rice and some kinds of white bread, and replace them with healthier choices. They perceived this requirement as particularly challenging because of different, compromised taste.

All participants were aware of the relationships between glucose intake, exercise and blood glucose level, and they knew when and how often to measure their blood glucose. They clearly understood the GDM self-management guidelines but found them hard to follow.

When I was pregnant, I had to measure [my blood glucose] before and after every main meal. That’s six times a day. And you know, that is a lot. I find it very cumbersome and energy consuming. Because one really has to remember. (participant 8)

Knowledge gained during pregnancy about the relationship between diet, exercise, blood glucose level and weight was potentially useful after delivery to prevent T2D. All participants were aware of the high risk of developing T2D later in life and how they could reduce this risk. However, they also described discontinuing the dietary recommendations after delivery and had generally allowed themselves to give in to food cravings. Participants considered the dietary regimen during pregnancy to be very strict; many expressed a need to relax restrictions after delivery and focus instead on their newborn.

… just after the delivery, I’ve noticed that I’m not as watchful as during the pregnancy. Because I know, OK, I don’t have diabetes, so I said to myself ‘well, well, I may be a bit unhealthy’, right? (participant 6)

I do go out for walks here and there, but I am not able to give a lot of time to exercising right now. I am mostly occupied with the baby right now. So I don’t get a lot of time to exercise. (participant 9)

Nevertheless, most participating women expressed the desire for a healthier lifestyle in the years after delivery, and many reported that they had continued some healthier food and exercise habits developed during pregnancy. Many women also expressed interest in participating in a post-delivery health intervention aimed at preventing T2D.

Although participants primarily described having GDM as a stressful and negative experience, some also perceived benefits. Some women who were overweight lost weight due to dietary modifications. Some experienced increased support from their husband and relatives during pregnancy, whereas others perceived the diagnosis as a wake-up call enabling them to act in ways that could delay or prevent development of T2D. Finally, some women gained more knowledge and insight into their bodies that could be useful for preventing T2D.

So, in a way, it has been good that I have had GDM. I kind of watch what I eat and don’t eat and know how I should stick to that. (participant 6)

But the funny thing was that I was also learning [something new] when I had gestational diabetes. Because when my blood sugar was … out of balance I felt like eating all sorts of sweets. But when it balanced, I lost the craving. And this I only first realized when I began eating healthy, and that is why it is now in balance. I did not know that earlier. (participant 2)

One participant also emphasised the importance of comprehensive communication and advice when instructing immigrants in GDM management guidelines and the use of related medical equipment.

If you come from another country, and did not grow up here, because those who grew up here, they know everything. … . But they [the health care professionals] showed me what to do. Now I have done exactly as they told me. (participant 5)

Participants gained valuable knowledge and experiences from their GDM self-management, which could potentially increase the likelihood of preventing future T2D. Knowledge and habits acquired during GDM management can contribute to the perception of risk and motivation for healthy living following delivery and long term. The regimented and stressful experience of managing GDM during pregnancy becomes a preview of what is to come and a warning against developing T2D. Although they found it challenging to make dietary and exercise changes, they generally expressed the desire for healthier daily lives and to take action to prevent T2D in the future.

#### 3.2.5 Contextual challenges

Some participants faced challenges directly linked to their immigrant status. One was separation from close relatives, but the use of social media allowed most women to be in daily contact with close relatives living in their countries of origin or elsewhere. Another and more devastating challenge directly linked to some participants’ immigrant status was trauma from war. Three women traumatised by war experiences described living with terrifying memories of air raids and loved ones being killed or kidnapped.

And especially when I watch television, such a similar situation, then I begin … then I start crying without any reason. (participant 1)

Not only were participating women traumatised – so were their relatives. For instance, one participant’s husband was so traumatised that she had to help him perform basic daily activities like getting out of bed in the morning.

As a child, another participant had spent a year in prison with her mother in her country of origin because her brother fought against the government and was subsequently separated from the family for an extended period of time. To some degree, the trauma some participants had endured influenced their perception of other challenges, like the risk of developing T2D. Coping with such extreme experiences could make this risk seem less important.

Participants experienced challenges related to their cultural backgrounds less often. One had been in two arranged marriages and now felt betrayed by both ex-husbands, who she felt had misused her to gain access to living in Denmark. In her most recent marriage, her husband left her when she was pregnant with their child. Shortly after, she was diagnosed with GDM.

Eeehhh, I was also, well, I was tired in the end and felt inflated. It was tough, you know, to sit alone with the worries, and he had just walked away, and now I sat all alone with the bills and the like, and a four-year-old kid. And if you are heavily pregnant, then … I remember, it really wore me down. That was what occupied me rather than GDM. (participant 10)

Most participating women were burdened by other challenges that affected their mental wellbeing and available social support. In some cases, depression resulted. Because accessing professional help is a taboo in some cultural environments, it became even more challenging.

(…) in our [cultural] environment, it is not well received, when you say ‘she’s got depressed’ or ‘she sees a psychologist’. They actually perceive it very negatively. Therefore, I dońt tell any of my [acquaintances] … My sisters know that I see a psychologist. But my father and my brothers dońt know that, and I dońt want to tell them. (participant 2)

Some participating women also suffered from other challenges, such as illness, painful physical conditions, lack of time or poor finances, that were not directly linked to their immigrant status or cultural background. These challenges were sources of worry and could also have a more direct effect on participants’ options for preventing T2D. For instance, one woman emphasised the high cost of healthy food and another quit her membership at a local fitness centre due to lack of money.

## 4. Discussion

### 4.1 Key findings

This study explored the perceptions of risk and motivation for healthy living among immigrants from non-western countries with prior GDM. We identified five main themes: (1) aetiology, (2) symptoms, (3) risk and prognosis, (4) management and pathophysiology, and (5) contextual challenges.

Most participants perceived their GDM diagnosis to be a result of genetic predisposition and poor diet. One also mentioned a connection between T2D and God’s will, a more fatalistic view. Overall, participating women’s view of aetiology was very much in keeping with the clinical explanation at the hospitals, which distinguishes our findings from those of previous studies. A study among migrant African women with GDM living in Sweden indicated low risk awareness and limited knowledge of GDM (Hjelm, Bard, & Apelqvist, 2018). Two other Swedish studies found that women with prior GDM perceived God’s intervention in health matters as important, both among Somali migrants (Wallin & Ahlström, 2010) and group of participants of mixed origin (Hjelm, Bard, Nyberg, & Apelqvist, 2003).

Generally, women with GDM found it hard to tell which symptoms were due to their diagnosis and which were related to pregnancy *per se*. This is consistent with a study among Pima Indian women with GDM in the United States (Smith-Morris, 2005).

Women participating in our study had experienced the consequences of severe T2D among their relatives. Although they expressed concerns about the health of their baby, their main worry during and after pregnancy was the risk of developing T2D and how it could affect their ability to take care of their children. Most participants had a family history of T2D. They were well aware of the risk of future T2D and even had a sense of its inevitability because of their family history. In contrast, in an Australian study, South Asian immigrant women with prior GDM were mainly concerned about the health of the baby (Bandyopadhyay et al., 2011). The same was true for women of Danish ethnicity living in Denmark (Svensson, Nielsen, & Maindal, 2018), which may be due to fact that they were less likely to have relatives with T2D who had experienced serious or fatal consequences. The latter seems likely; past experiences with severe T2D among relatives had a strong influence on the perception of risk and motivation for healthy living among participants in our study. They showed strong risk awareness, as opposed to the findings of two Swedish studies by Hjelm and colleagues (Hjelm et al., 2018; Hjelm, Bard, Berntorp, & Apelqvist, 2009). In a study of perceptions about postpartum health and illness among women born in Sweden and the Middle East, Hjelm et al. found less risk awareness among immigrant women than among those born in Sweden (Hjelm et al., 2009). In contrast, ethnic minority women from Canada were more likely to report improved health behaviour, like reducing portion sizes and increasing physical activity, after a diagnosis of GDM than Caucasian women were (Banerjee et al., 2016).

Women participating in our study were taught how to measure and regulate their blood glucose levels during pregnancy. They generally understood these methods well and, even though GDM self-management was demanding, were able to regulate their blood glucose levels. They found it difficult to maintain the health behaviour changes after birth. This finding is consistent with findings of a study in Australia (Bandyopadhyay, Small, & Davey, 2015). In contrast, a Danish study among immigrants with GDM found that hospital-based information was sometimes misunderstood (Dayyani, Terkildsen Maindal, Rowlands, & Lou, 2019). Similarly, the Australian study among South Asian immigrants revealed that their perceptions of healthy eating and exercise habits during pregnancy with GDM contrasted with biomedical explanations, e.g. restricted diets would prevent growth of the baby and exercise would put too much strain on the baby (Bandyopadhyay et al., 2011).

Finally, our study indicates that when a disease during pregnancy is accompanied by other psychosocial and medical challenges, women experience additional pressure that makes management or prevention more challenging. Other studies confirm this finding. For instance, an Australian study by Carolan and colleagues found that physical constraints and difficulty finding the time required inhibited GDM self-management among women of immigrant or disadvantaged backgrounds (Carolan, Gill, & Steele, 2012). A study of experiences of GDM among East African immigrant women in Canada found that financial barriers were the primary challenge to diabetes management (Siad et al., 2018), and a study of Korean immigrants in the United States at risk for T2D found that negative emotions and depressive symptoms may be related to behavioural risk factors (Choi, Rush, & Henry, 2013).

### 4.2 Construction of illness

According to Kleinman (1980), construction of illness experiences is the earliest health care function and is often a personally and socially adaptive response. The construction of illness from disease is a coping function and the first stage of healing, occurring even before proper treatment is obtained (Kleinman, 1980). Such illness constructions are essential to preventive and curative care, particularly during a pregnancy with GDM. After pregnancy, illness experiences may still have a strong influence on motivation for healthy living, even though some factors that were part of the construction of illness during pregnancy are no longer as relevant, e.g. fear of harming the baby in the womb.

Symptoms constitute an important part of illness construction. Yet, in the case of GDM, symptoms were usually absent. Moreover, although symptoms of GDM were hard to detect, T2D complications were very evident among participants’ relatives and experiences of T2D-related deaths and amputations were not uncommon. Participating women found motivation for GDM management during pregnancy and healthy living after pregnancy due to their primary GDM-related concern of developing T2D. Thus, experiences with severe cases of T2D among relatives affected the perception of risk and motivation for healthy living.

Even though participating women perceived their pregnancies with GDM as negative experiences, most also experienced positive outcomes from the condition, such as weight loss and increased knowledge about their own bodies. These positive experiences also contributed to the construction of illness and potentially facilitated healthy lifestyle choices.

### 4.3 Experiences and human suffering can affect healthy living

Although some healthy behaviours adopted during pregnancy were maintained after delivery, participants’ experiences of and struggles with adopting them may have negatively affected the priority they gave to healthy behaviours and their perceptions of risk and motivation for achieving a healthier lifestyle.

Kleinman’s (1980) explanatory model does not include the theme of contextual challenges that emerged from our data. However, his later work (1995) focuses on experiences and human suffering. When analysing various aspects of illness, Kleinman stresses the importance of taking individuals’ experiences into account and developing a more nuanced understanding of their sufferings.

Many women participating in our study were suffering due to war experiences, longing for relatives in their country of origin, poverty, confusion about cultural and social customs in their new country, lack of time, social control and painful physical conditions. Some were deeply affected by their experiences, and their suffering made their perceived risk for T2D development less important.

### 4.4 Vulnerability and culture

In one sense, study participants were vulnerable because their diagnoses of GDM were accompanied by other challenges. However, they also demonstrated great resilience and the capacity to overcome very challenging situations, e.g. fleeing from a war-torn country. Participants described some challenges as linked to their immigration status, but they very rarely linked challenges to their cultural backgrounds. Thus, they were vulnerable, although the challenges were related to immigration and broader socio-economic issues rather than their cultural backgrounds *per se*. In many ways, calling study participants vulnerable simplifies a complex state of affairs. Most participants displayed significant psychological strength to overcome severe challenges and remain standing.

### 4.5 Implications for practice and research

For health promotion to be effective and have a sustainable impact, it must take the life worlds of target groups into consideration, including perceptions and contextual situations (Green, Cross, Woodall, & Tones, 2019). Our study addressed perceptions of risk and motivation for healthy living among a group of women at particularly high risk of future T2D who also faced a range of other challenges. Based on our findings, we recommend that health promotion interventions among immigrant women with non-western backgrounds at high risk of developing T2D take their illness constructions of GDM as a starting point.

In addition, participants had first-hand experiences with possible adverse consequences of T2D among their relatives, which stimulated their motivation to prevent T2D by adopting healthy behaviours. This motivation is essential for successful health promotion and should be encouraged and further stimulated by health professionals.

Furthermore, challenges that study participants faced on a daily basis had a critical impact on their available resources for healthy behaviours. Some challenges were so profound that they warranted being addressed as an integrated part of health promotion. Future health promotion efforts with non-western immigrant women should address health in a holistic way by integrating mental and social health into interventions aimed at preventing diabetes. This perspective is essential for health care practitioners to provide meaningful and effective health promotion interventions.

Major differences exist between immigrant groups across countries. Consequently, research is needed to identify local variations in risk perceptions and motivation levels to facilitate public health planning.

### 4.6 Strengths and limitations

The study provides evidence related to an important but under-researched area of public health. At the same time, several methodological limitations deserve mention.

In 2018, the employment rate was 57% among immigrant women with non-western backgrounds in Denmark (Statistics Denmark, 2019), but most women who participated in interviews were employed. Participants may have been more acculturated and settled in Denmark and had a higher socio-economic status than average immigrant women. To address this limitation, some interviews were conducted with women who were diagnosed with GDM several years ago and struggled with unemployment and other challenges at that time. At the time of interviews, they had more mental and emotional reserves, were employed and had sufficient resources to participate in the study. Had we asked them to participate during the earlier period, they may have declined, but they were able to share their stories several years after their previous GDM pregnancy. Study recruitment proved somewhat difficult, and twelve informants constitute a fairly small sample. However, we achieved good variation in participants’ ethnicity, age and education and reached data saturation after 17 interviews. Participants’ duration of residence in Denmark did not make any difference to their risk perception nor motivation for healthy living. However, a larger sample size might have enabled us draw firmer conclusions.

About half of the interviews were conducted in clinical settings, which may have influenced participants’ responses toward biomedical views. To minimise this risk and foster an open dialogue, the interviewer (SB) stressed her professional background as an anthropologist with no medical training or affiliation with the hospital. Furthermore, the interviewer was a female of Danish descent and reproductive age. Her gender and age may have helped establish rapport with participating women. Cultural differences between researcher and participants could have been a barrier to communication, but the interviewer attempted to minimise this challenge by dressing appropriately for participants’ cultural customs and acknowledging any cultural differences they expressed. Conversely, the researcher may have benefitted from cultural differences because her status as an outsider mitigated any concerns that participants’ responses would be shared within their ethnic and cultural communities despite assurances of confidentiality.

## 5. Conclusion

Among immigrant women from non-western countries with prior GDM living in Denmark, several factors influenced their perceptions of risk and motivation for healthy living. From a public health perspective, our findings are highly relevant because GDM is an important risk factor for developing T2D later in life. There is strong evidence supporting behavioural changes, but the circumstances of a newborn in the family and frequent competing social and health challenges can make such changes difficult to achieve. The findings contribute to an understanding of the perceptions and living conditions of immigrant women that are vital to tailoring support and interventions to prevent later development of T2D.
